# Role of Sintering Temperature in Production of Nepheline Ceramics-Based Geopolymer with Addition of Ultra-High Molecular Weight Polyethylene

**DOI:** 10.3390/ma14051077

**Published:** 2021-02-25

**Authors:** Romisuhani Ahmad, Mohd Mustafa Al Bakri Abdullah, Wan Mastura Wan Ibrahim, Kamarudin Hussin, Fakhryna Hannanee Ahmad Zaidi, Jitrin Chaiprapa, Jerzy J. Wysłocki, Katarzyna Błoch, Marcin Nabiałek

**Affiliations:** 1 Faculty of Mechanical Engineering Technology, Universiti Malaysia Perlis (UniMAP), P.O. Box 77, D/A Pejabat Pos Besar, Kangar, Perlis 01000, Malaysia; wanmastura@unimap.edu.my (W.M.W.I.); kamarudin@unimap.edu.my (K.H.); fakhryna0508@gmail.com (F.H.A.Z.); 2 Center of Excellence Geopolymer and Green Technology, School of Materials Engineering, Universiti Malaysia Perlis (UniMAP), P.O. Box 77, D/A Pejabat Pos Besar, Kangar, Perlis 01000, Malaysia; mustafa_albakri@unimap.edu.my; 3 Synchrotron Light Research Institute, Muang, Nakhon Ratchasima 3000, Thailand; jitrin@slri.or.th; 4 Department of Physics, Częstochowa University of Technology, 42-201 Częstochowa, Poland; katarzyna.bloch@wip.pcz.pl (K.B.); nmarcell@wp.pl (M.N.)

**Keywords:** geopolymer, ceramics, nepheline, lightweight, sintering

## Abstract

The primary motivation of developing ceramic materials using geopolymer method is to minimize the reliance on high sintering temperatures. The ultra-high molecular weight polyethylene (UHMWPE) was added as binder and reinforces the nepheline ceramics based geopolymer. The samples were sintered at 900 °C, 1000 °C, 1100 °C, and 1200 °C to elucidate the influence of sintering on the physical and microstructural properties. The results indicated that a maximum flexural strength of 92 MPa is attainable once the samples are used to be sintered at 1200 °C. It was also determined that the density, porosity, volumetric shrinkage, and water absorption of the samples also affected by the sintering due to the change of microstructure and crystallinity. The IR spectra reveal that the band at around 1400 cm^−1^ becomes weak, indicating that sodium carbonate decomposed and began to react with the silica and alumina released from gels to form nepheline phases. The sintering process influence in the development of the final microstructure thus improving the properties of the ceramic materials.

## 1. Introduction

Inorganic non-metallic materials, so-called ceramics, consist of metallic and non-metallic elements bonded via ionic and/or covalent bonds. It can be polycrystalline or at least partly polycrystalline structure, formed by a sintering process [1]. Relative to traditional ceramics, various applications for advanced ceramics have seen many applications and the development for more advanced applications is growing at an equitable rate [2]. Generally, different types of ceramics originate with different properties such as low thermal conductivity; chemically inert; and high compression strength, modulus, and hardness [3].

The factor such as materials selection, binder addition, appropriate fabrication methods, and sintering profile must be accounted to meet the required properties and performance.

The demand for specific properties such, as better stiffness, stronger and lightweight ceramic materials recently generate a large interest in the ceramic processing industry. Commonly, types of additives used in ceramic processing consist of binder, plasticizers, surfactants, dispersants, and lubricants [4,5]. Polymer binder is usually used in ceramic processing which assists the primary function of providing strength to the green ceramic body.

Geopolymers are typically made by mixing materials containing aluminosilicate into alkali solutions and curing the mixture at a specific temperature [6,7,8,9]. Any materials that contain mostly silica and alumina can be used as source material in the production of geopolymer. These can be industrial byproduct materials such as fly ash, ground granulated blast furnace slag (GGBFS), red mud, silica fume, and rice hush ash [10,11,12,13]. On the other hand, natural minerals such as kaolin and metakaolin could also be used as source materials. Highly alkali solutes, such as sodium hydroxide (NaOH) and potassium hydroxide (KOH), are mixed into the source materials that are rich in SiO_2_ and Al_2_O_3_, resulting in Si–O–Al–O bonds. An alternate way of using geopolymer technology was found to decrease the high temperature required in producing ceramic materials. The amorphous to semicrystalline phase of geopolymers can be transformed into crystalline ceramic phases via sintering [1,14,15,16].

Kriven et al. (2013) reported that the fabrication of conventional ceramic materials requires particularly high temperature treatment of up to 1600 °C [17]. To overcome the problem, the geopolymer method can be used in fabricating high-performance lightweight ceramics at a slightly lower temperature with the intention of reducing the energy used towards environmental issues. According to Xie et al., the sintering of geopolymer above 1000 °C was sufficient to consolidate the sample and begin leucite crystallization. Heating to 1200 °C for 3 h resulted in a maximum in density, fracture toughness, and biaxial flexure strength [18], while Peigang et al. found that the crystallization peak temperature of K_2_O·Al_2_O_3_·5SiO_2_ was 986.3 °C and the temperature range of sintering was 700 °C to 954.3 °C [19]. The geopolymerization reaction will minimize the need for high-temperature techniques or processes to obtain materials with ceramic-like structure and properties [18,19].

High temperatures are essential in solid-phase sintering for facilitated diffusion. The profiles of the sintering mechanism promote material densification, while diffusion act as a mechanism that encourages densification and grain growth [20]. The sintering profile will permit densification to occur without stimulating the grain growth, which is suitable for microstructural development as the engineering properties of ceramic materials are dictated by the microstructure such as density and grain size. Sintering curve control for the manipulation of the microstructure during sintering is a route that has been discovered and offers advantages such as uncomplicated and economically feasible [21]. Thus, this paper elucidates the influence of sintering on the properties and microstructure of the nepheline ceramic produced using the geopolymer method. Ultra-high molecular weight polyethylene (UHMWPE) was used as the binder to produce lightweight nepheline ceramics.

## 2. Materials and Methods

### 2.1. Materials

The raw materials used to produce nepheline ceramics based geopolymer are kaolin, sodium hydroxide (NaOH), and sodium silicate (Na_2_SiO_3_). With a chemical composition of Al_2_Si_2_O_5_(OH)_4,_ (Table 1), kaolin consists of fine particles in the range of 0.7 µ to 150 µ, were supplied by Associated Kaolin Industries Sdn. Bhd. Selangor, Malaysia. The caustic soda micropearls of sodium hydroxide (NaOH) (purity 99%) were produced in Taiwan with a brand named Formosoda-P, and the sodium silicate (Na_2_SiO_3_) with a chemical composition of 30.1% of SiO_2_, 9.4% of Na_2_O, and 60.5% of H_2_O (modulus, SiO_2_/Na_2_O = 3.2) were supplied by South Pacific Chemicals Industries Sdn. Bhd. (SPCI), Pahang, Malaysia. The powder form of ultra-high molecular weight polyethylene (UHMWPE), with a molecular weight of 5 × 106 g/mol and a density of 0.94 g/mL was used as binder in geopolymer ceramics, and it was purchased from Ticona Engineering Polymer, Shanghai, China.

### 2.2. Experimental Procedures

The alkali activator was prepared by mixing the 12 M NaOH solution with Na_2_SiO_3_ at a ratio of 0.24, then stirring the mixture until it becomes clear. The solution was then aged for 24 h to allow it to fully dissolve. The paste of geopolymer was prepared by activating the raw material kaolin with alkaline activator solution at a solid-to-liquid ratio of 1.0. Then, the mixture was stirred until homogeneous using a mechanical mixer (TOHO, Selangor, Malaysia), then poured into a HDPE mold. The samples were then placed in an oven at 80 °C for 24 h to cure. Using the mechanical crusher (JHL, KL, Malaysia), the geopolymer was crushed and sieved using 150 microns siever to obtain fine powder. The geopolymer powder was then blended with 4 wt.% of UHMWPE in a dry condition process using a planetary mill (Corston, UK). The percentage of the binder was selected based on previous works, as it resulted in optimal mechanical and thermal properties. To produce a homogeneous mixture, the mixing process was performed for 4 min at 100 rpm in the reverse direction.

The mixture was then compacted with a cylindrical stainless-steel die (Ipoh, Perak, Malaysia) of 12 mm in diameter at 5 tons for 2 min, and the final green body was sintered on an alumina plate at 900 °C, 1000 °C, 1100 °C, and 1200 °C in an electrical furnace (LT Furnace Model HT4-1600-SIC, (Shanghai, China) in air atmosphere, with a soaking time of 180 min of and a heating rate of 5 °C/min. 

### 2.3. Physical and Mechanical Testing

The mechanical properties of the nepheline ceramics-based geopolymer were determined in terms of flexural strength. The three-point bending fixture was used to test the specimens at dimensions of 7 mm × 5 mm × 52 mm. The distance of the support span was 30 mm and a crosshead speed of 0.3 mm/min was used. The theoretical density and porosity of nepheline ceramic samples were determined using a pycnometer (AccuPyc II 1340 He pycnometer, Micromeritics, GA, USA). The volumetric shrinkage of the samples was manually measured, and the water absorption test was conducted based on the ASTM C373-88 to determine the absorbed water ability of the samples upon immersion in water [22].

For the correlation study, the data from two variables were plotted into a linear graph. A linear equation was calculated using software and positive correlation indicates that either variable increase or decrease together, whereas negative correlation indicates that one of the variables increases and the other decrease. Pearson’s correlation coefficient (R^2^) was used to measure the strength of the association between the two variables. In terms of the strength of the relationship, the value of the correlation coefficient varies between +1 to −1. 

### 2.4. Thermal Analysis

The weight change over temperature ranges indicates the degradation composition of the sample and thermal stability. The thermal analysis was carried out using a Pyris Diamond TGA from Perkin Elmer (Midland, ON, Canada) The samples weight (7 ± 2 mg) underwent thermal scanning over a temperature range from 30 °C to 1000 °C using a nitrogen air flow of 50 mL/min and a heating rate of 10 °C/min.

### 2.5. Microstructural Characterization Testing

The morphological analyses were conducted using scanning electron microscopy (SEM) equipped with secondary electron detectors. JSM-6460LA model Scanning electron microscope (JEOL, Peabody, MS, USA) was used to image the microstructure of the nepheline ceramic samples. Auto Fine Coater, model JEOL JFC 1600 (Peabody, MS, USA) was used to coat the samples before imaging.

X-ray diffraction (XRD) data were obtained using XRD 6000, SHIMADZU diffractometer (OR, USA). The samples were prepared by pressing the powder samples into aluminium holders. The operating conditions were 40 kV and 30 mA and performed using Cu-Kα radiation in the range 2θ value of 10° to 80°. The X’Pert High Score Plus software (Version 2.0) was used for peak identification, and the automated search match was used to analyze the diffraction patterns.

The chemical bonds within the samples were determined using a Perkin Elmer Spectrometer 2000 Fourier Transform Infrared (FTIR) Spectroscopy (Akron, OH, USA). The potassium bromide (KBr) pellet technique was used with the ratio of specimen-to-KBr is 1:100. 300 mg of KBr and 3 mg of powdered specimen were put into mold and pressed using a cold press machine at 4 tons for 2 min. The pellet was then scanned from a range of 450 cm^−1^ to 4000 cm^−1^ and the resolution for all the infrared spectra was 4 cm^−1^. The spectrum was collected after the background spectrum was substrates.

**Figure 1:**
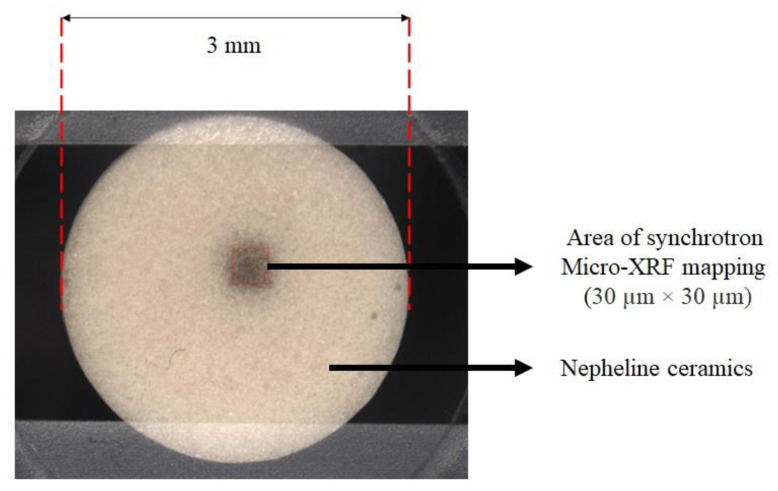
Specification of nepheline ceramic based geopolymer with the addition of ultra-high molecular weight polyethylene (UHMWPE) sample for synchrotron micro-X-ray fluorescence (µ-XRF).

The distribution of elements in kaolin-based geopolymer and nepheline ceramics were evaluated using synchrotron µ-XRF at BL6b beamline at the Synchrotron Light Research Institute (SLRI), Nakhon Ratchasima, Thailand. A polycapillary lens to create a micro-X-ray beam with a beam size of 30 µm × 30 µm on the samples was amplified by continuous synchrotron radiation as shown in Figure 1. The testing was performed with an exposure time of 30 s for each stage in the helium gas atmosphere. The beam size range used in the facilities was 2–20 keV and the result was clarified and analyzed using PyMca software (Version 5.6.3).

## 3. Results

**Figure 2:**
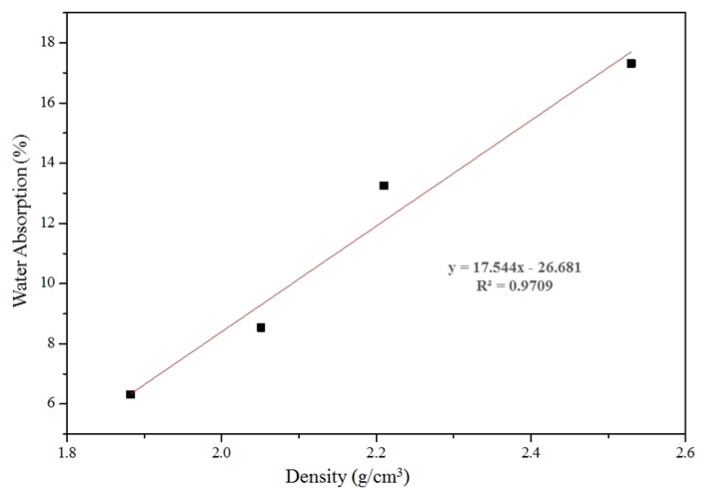
The relationship between water absorption and density of nepheline ceramics-based geopolymer with the addition of UHMWPE at sintering temperature of 1200 °C.

The physical properties of the nepheline ceramics-based geopolymer with the addition of 4 wt.% UHMWPE such as flexural strength, density, porosity, volumetric shrinkage, and water absorption were evaluated at four sintering temperatures, and the results were compiled in Table 2. The increased sintering temperature influences the physical and mechanical properties of nepheline ceramics-based geopolymer. The results show that the highest flexural strength of 92 MPa was achieved once the samples are used to sinter at 1200 °C, attributed to the formation of a smooth matrix due to the development of a large sintered area permitted by the well diffusion of the sample particles [23]. The increasing of carbon content in the geopolymer system attributed to the decomposition of UHMWPE also leads to increase in the flexural strength. The theoretical density of the samples demonstrated an inverse relationship with the sintering temperatures. Samples sintered at 900 °C had the highest density of 2.53 g/cm^3^, which decreased to 2.21 g/cm^3^ at 1000 °C, then gradually decreased to 1.88 g/cm^3^ at 1200 °C. The decreasing of density can be attributed to the liquid sintering and decomposition of UHMWPE that leads to the closure of accessible pores and pore channels [24]. The correlation from the effect of sintering temperature on the kaolin-based geopolymer with the addition of UHMWPE indicates a strong relationship (Figure 2). The correlation between water absorption and density shows the highest R^2^ value of 0.9709, which is expected as sintering created pores that lead to decreasing the density of the samples. The equation governing this relationship is y = 17.544x − 26.581, where y is the water absorption (%), and x is the density (g/cm^3^).

**Figure 3:**
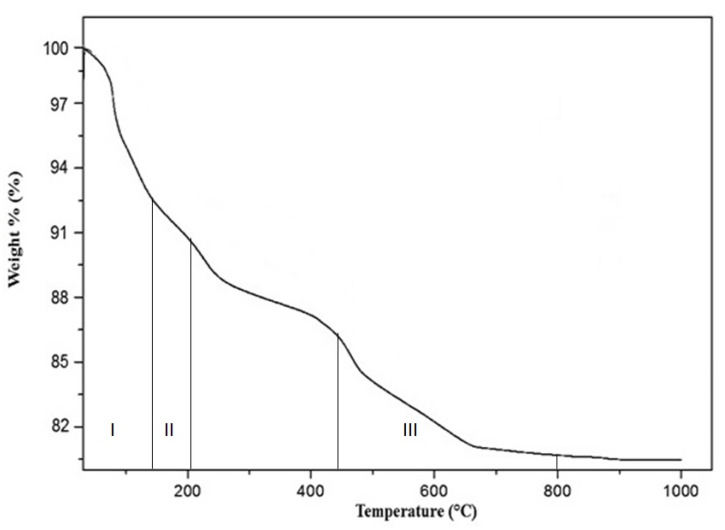
Thermal Gravimetry Analysis (TGA) curve of kaolin based geopolymer with the addition of UHMWPE.

The thermal gravimetry (TG) results of kaolin based geopolymer with the addition of UHMWPE as binder upon heating are presented in Figure 3. The first major weight loss was associated with the dewatering process observed in the temperature range of 0 to 120 °C. The gradual weight loss observed to 300 °C could be owed to the evaporation of free water including unconstrained surface water and pore or the water micro-capillaries inside the samples [19]. Similar weight loss has been observed in other investigations on Na-, K-, and Na/K-based geopolymer [1,14,19]. The weight loss before 700 °C of geopolymer has been reported that it might be obtained from the water loss, by evaporation of both free water and condensed hydroxyl groups. When the temperature further increased, the kaolin-based geopolymer mixed with UHMWPE exhibited little weight change and reaches a plateau at around 700 °C. In the addition of the UHMWPE case, the weight loss was just not caused by the elimination of water only. The gradual decomposition of UHMWPE continues over a large range of temperature also present a rapid thermal degradation. There is a considerable loss in weight is significant once the temperature achieves 800 °C, this loss in weight continuing until higher temperature 1000 °C with a light change in weight. 

**Figure 4:**
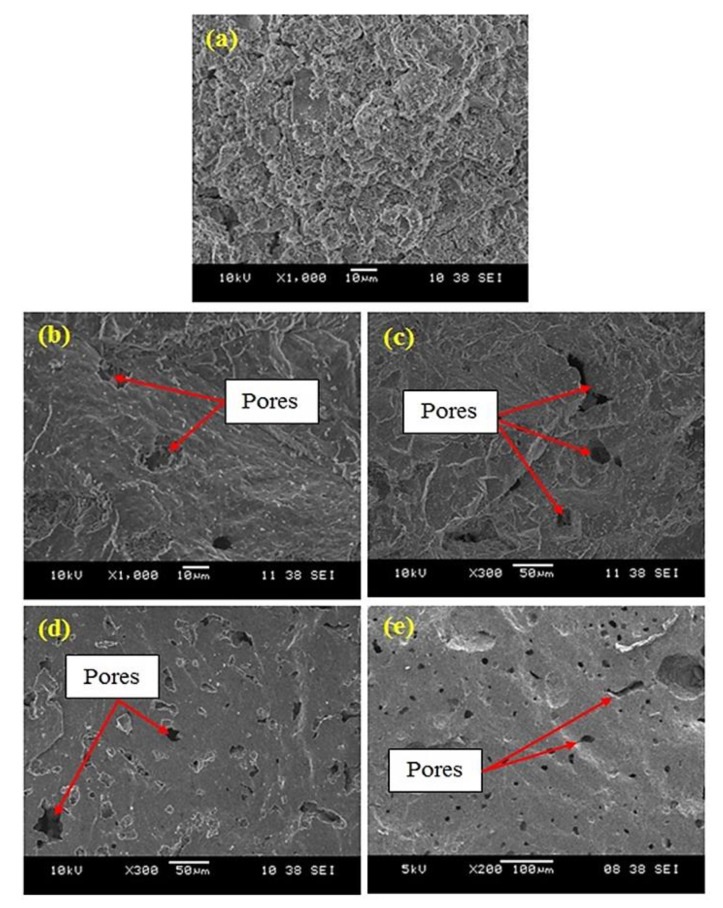
SEM micrograph of (**a**) kaolin-based geopolymer and nepheline ceramics based geopolymer with addition of UHMWPE at different sintering temperatures (**b**) 900 °C, (**c**) 1000 °C, (**d**) 1100 °C, and (**e**) 1200 °C.

The SEM micrographs of a fracture surface of kaolin-based geopolymer and nepheline ceramics-based geopolymer with the addition of UHMWPE at sintering temperatures of 900 °C, 1000 °C, 1100 °C, and 1200 °C is shown in Figure 4. In the microstructure of kaolin-based geopolymer, a continuous gel-like region and some porous distribution have been found, suggesting that the pure geopolymer binder enhances the structure itself and contributes to increasing the strength [25]. The sintering of geopolymer consequently resulted in the formation of a liquid phase, which allows the joining of particles and the transformation of plate-like structure into a denser microstructure. For the samples sintered at 900 °C and 1000 °C, a sponge-like gel texture is evident. A relatively well-developed microstructure resulted in a more compact and smoother surface as the samples were sintered to a higher temperature of 1100 °C and 1200 °C. The high sintering temperature sustained the consolidation and aiding a fairly uniform microstructure, thus creating a smooth surface texture. The sintering process induced densification via the elimination of the finest particle, the pore size becomes irregular and some of pores were confined within the grains [26,27]. The presence of small pores throughout the sample represents the transformation of the amorphous phase to the nepheline phase of crystalline ceramic, confirming that sintering primes phase transformation [28]. Besides, the addition of UHMWPE also resulted in the formation of pores through the gradual decomposition process during sintering, and the results were supported by the increase of the flexural strength and decrease in the density of the samples. 

**Figure 5:**
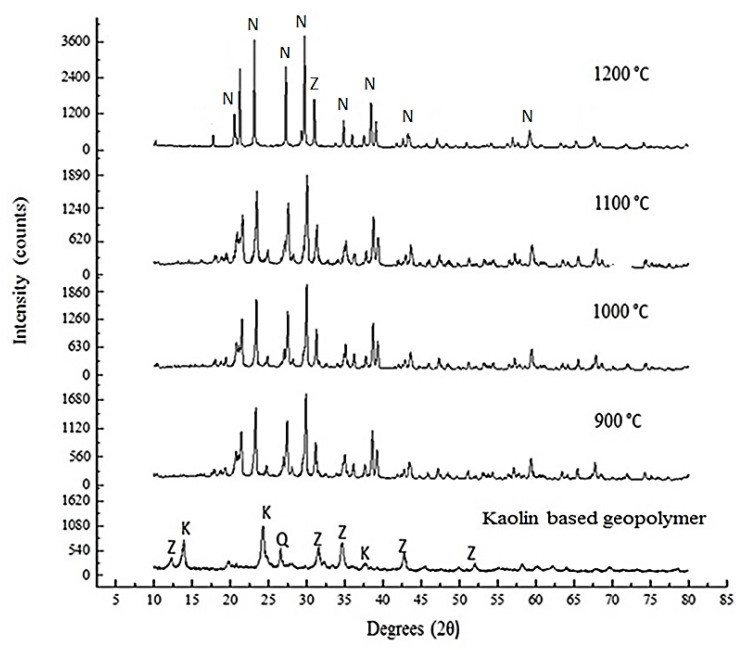
XRD pattern of kaolin-based geopolymer and nepheline ceramics-based geopolymer with the addition of UHMWPE at different sintering temperatures (N = Nephaline ICDD# 35-0424, K = Kaolinite ICDD# 29-1488, Q = Quartz ICDD# 46-1045, Z = Zeolite ICDD# 30-0238).

Figure 5 illustrates the changes of XRD pattern in the amorphous phase of the kaolin-based geopolymer during sintering. The appearance of zeolite (Z) at 2θ value of 12.4°, 31.6°, 35.0°, 43.8°, and 52.9°; kaolinite (K) at 14.5°, 24.2°, and 37.8°; and quartz (Q) peaks at 26.5° were found in kaolin based geopolymer. The kaolinite (Al_2_SiO_5_(OH)_4_) and quartz (SiO_2_) were the mineralogical components of kaolin, while the zeolite is typically crystallized from the formation of amorphous aluminosilicate gel formation by the activation of kaolin with the alkali activator. The crystallographic composition was changed as the hump of the amorphous phase disappeared during the heat treatment due to the phase transformation from amorphous to crystalline [28]. The samples sintered at 900 °C to 1200 °C show very comparable peaks with the appearance of the same nepheline (NaAlSiO_4_). The peaks became narrower as the temperature increased. The new phase corresponding to nepheline was detected, in line with the use of sodium-based activator in geopolymer synthesis. However, the intensity of the nepheline peaks increased with increasing sintering temperature, which indicates that the increased crystallinity consequently improved the mechanical strength of the samples [29]. 

**Figure 6:**
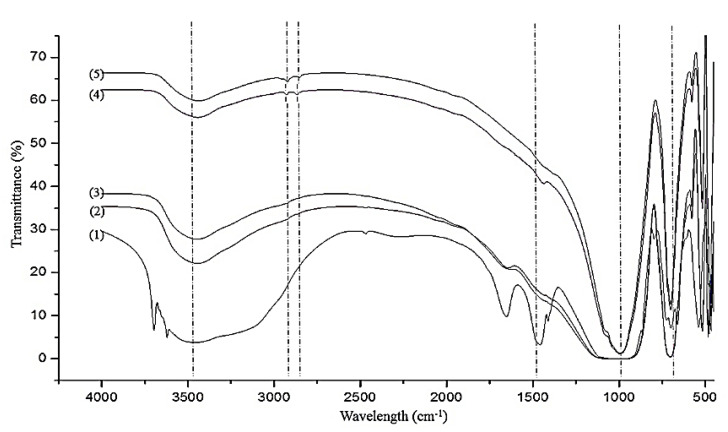
IR spectra of (**1**) kaolin-based geopolymer and nepheline ceramics-based geopolymer with the addition of UHMWPE sintered at (**2**) 900 °C, (**3**) 1000 °C, (**4**) 1100 °C, and (**5**) 1200 °C.

The IR spectra of kaolin-based geopolymer and nepheline ceramics based geopolymer with the addition of UHMWPE sintered at different temperatures are shown in Figure 6. The kaolin-based geopolymer spectrum includes absorption bands corresponding to O-H vibration at 3696 cm^−1^, 3620 cm^−1^, and 1650 cm^−1^, indicating that H_2_O bond was absorbed into the structure or caught in the structural cavities. A broad absorption band at approximately at 1450 cm^−1^ is related to the asymmetric stretching of carbonate (CO_3_^2−^) ions due to inducing atmospheric carbonation of alkali metal hydroxide and the gels [30]. The strongest vibration centered is roughly at 1000 cm^−1^, referred to as a major geopolymer fingerprint, which is the asymmetric stretching of Si–O–Si (Al) bridges. 

The FTIR measurements is basically in accordance with the results of XRD and proved that the nepheline phase appeared when the samples were heated. The appearance peak at 700 and 1040 cm^−1^ corresponds to the Si–O–Si groups, while the peaks at 2842 cm^−1^ and 2931 cm^−1^ corresponds to the C–H asymmetric stretching vibration due to the addition of UHMWPE as a binder in the geopolymer system. The OH stretching band at around 3400 cm^−1^ becomes weak after the sintering due to dehydration and dehydroxylation process, while the OH-bending bond at around 1650 cm^−1^ was absent after sintering at 1100 °C and 1200 °C. A presence band at around 1400 cm^−1^ can be attributed to the asymmetric stretching of CO_3_^2−^ [31]. The band at 1450 cm^−1^ becoming weak once it is sintered and vanished at 1200 °C, indicating that the sodium carbonate decomposed and starts to react with the silica and alumina released from gels to form nepheline.

**Figure 7:**
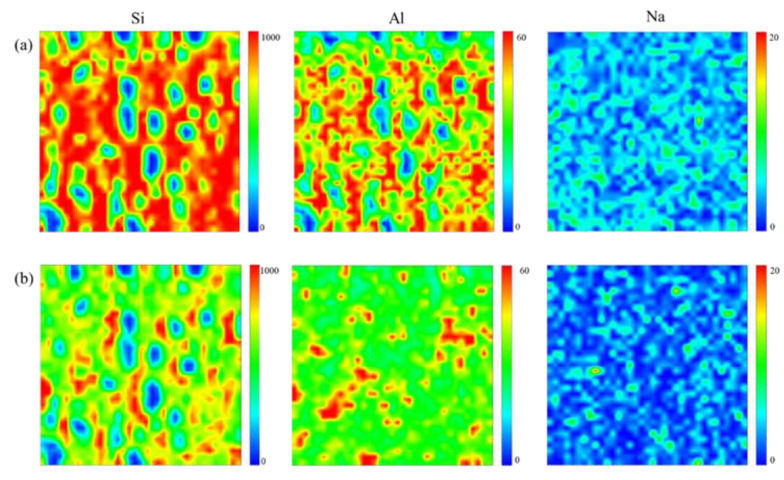
Micro-XRF elemental distribution maps of Na–Al–Si for (**a**) kaolin-based geopolymer (**b**) nepheline ceramics based geopolymer with the addition of UHMWPE at 1200 °C sintering temperature.

Figure 7 shows the results of synchrotron micro-XRF elemental mapping in order to determine the distribution of Si, Al, Na in kaolin-based geopolymer and nepheline ceramics-based geopolymer. The samples sintered at 1200 °C were selected for micro-XRF analysis due to the well-densified microstructure images. The elemental distribution represents the minerals that can be precisely localized through the result obtained within the alkali activated structure. The colors blue, green, and red reflect each distribution factor for low, medium, and high intensities in the integrated area. The high concentration of Si and Al region for kaolin-based geopolymer identified the alkali activated backbone (Si–O–AL/Si). For the sample sintered at 1200 °C, the high concentration and good distribution of Si, Al, and Na elements is indicative of the presence of the nepheline phase.

## 4. Conclusions

In conclusion, the nepheline ceramics based geopolymer were successfully fabricated using geopolymer method at 900–1200 °C. The role of sintering on the physical, mechanical, and microstructural properties of nepheline ceramics-based geopolymer with the addition of UHMWPE as a binder was investigated. A compacted and smooth surface microstructure with the highest strength of 92 MPa and lowest density of 1.88 g/cm^3^ were obtained at the sintering temperature of 1200 °C. Structural consolidation was achieved and resulted in a relatively uniform and smooth microstructure at higher sintering temperatures. The presence of pores shows a significant contribution to the improvement of properties, due to phase transformation occurring during the sintering process. The sintering profile will promote material densification while diffusion is the matter transport mechanism that promotes densification and grain growth. The sintering profile of geopolymer materials is important in understanding the effort to improve the mechanical and microstructure properties of geopolymer ceramics.

## Figures and Tables

**Table 1:** Composition of kaolin (wt.%).

Composition	SiO_2_	Al_2_O_3_	Fe_2_O_3_	K_2_O	TiO_2_	MnO_2_	ZrO_2_	LOI
Percentage (%)	54.0	31.7	4.89	6.05	1.41	0.11	0.10	1.74

**Table 2:** Physical and mechanical properties of nepheline ceramics based geopolymer with the addition of UHMWPE at different sintering temperatures.

Sintering Temperature	900 °C		1000 °C		1100 °C		1200 °C	
	Average	SD	Average	SD	Average	SD	Average	SD
Flexural Strength (MPa)	42.35	1.81	50.12	1.71	65.65	1.82	92	1.51
Theoretical density (g/cm^3^)	2.53	0.02	2.21	0.03	2.05	0.02	1.88	0.02
Total Porosity (%)	25.35	0.5	26.93	0.42	28.23	0.3	34.01	0.61
Volumetric Shrinkage (%)	10.02	0.62	13.54	0.32	16.55	0.54	18.32	0.63
Water Absorption (%)	6.32	0.63	8.54	0.32	13.26	0.51	17.31	0.63

SD, standard deviation.

## Data Availability

The data presented in this study are available on request from the corresponding author.
